# Periodic 17β-Estradiol Pretreatment Protects Rat Brain from Cerebral Ischemic Damage via Estrogen Receptor-β

**DOI:** 10.1371/journal.pone.0060716

**Published:** 2013-04-12

**Authors:** Ami P. Raval, Raquel Borges-Garcia, William Javier Moreno, Miguel A. Perez-Pinzon, Helen Bramlett

**Affiliations:** 1 Cerebral Vascular Disease Research Laboratories, Department of Neurology, University of Miami, Miami, Florida, United States of America; 2 Department of Neurological Surgery, University of Miami, Miami, Florida, United States of America; 3 Neuroscience Program, Leonard M. Miller School of Medicine, University of Miami, Miami, Florida, United States of America; 4 Bruce W. Carter Department of Veterans Affairs Medical Center, Miami, Florida, United States of America; Massachusetts General Hospital/Harvard Medical School, United States of America

## Abstract

Although chronic 17β-estradiol (E_2_) has been shown to be a cognition-preserving and neuroprotective agent in animal brain injury models, concern regarding its safety was raised by the failed translation of this phenomenon to the clinic. Previously, we demonstrated that a single bolus of E_2_ 48 hr prior to ischemia protected the hippocampus from damage in ovariectomized rats via phosphorylation of cyclic-AMP response element binding protein, which requires activation of estrogen receptor subtype beta (ER-β). The current study tests the hypothesis that long-term periodic E_2_-treatment improves cognition and reduces post-ischemic hippocampal injury by means of ER-β activation. Ovariectomized rats were given ten injections of E_2_ at 48 hr intervals for 21 days. Hippocampal-dependent learning, memory and ischemic neuronal loss were monitored. Results demonstrated that periodic E_2_ treatments improved spatial learning, memory and ischemic neuronal survival in ovariectomized rats. Additionally, periodic ER-β agonist treatments every 48 hr improved post-ischemic cognition. Silencing of hippocampal ER-β attenuated E_2_-mediated ischemic protection suggesting that ER-β plays a key role in mediating the beneficial effects of periodic E_2_ treatments. This study emphasizes the need to investigate a periodic estrogen replacement regimen to reduce cognitive decline and cerebral ischemia incidents/impact in post-menopausal women.

## Introduction

Strong evidence from experimental animal models suggests that chronic continuous 17β-estradiol (E_2_) treatment has both potent and long-lasting effects on improved pathophysiological outcome after brain ischemia [1]–[3]. This prompted the initiation of the Women’s Estrogen for Stroke Trial (WEST) and Women’s Health Initiative (WHI). The outcomes from these trials were, however, unsuccessful in showing any benefits of hormone replacement therapy and also raised questions about the safety of chronic E_2_ treatment and the timing of initiation of estrogen therapy in women [4], [5].

To address the safety of continuous chronic E_2_ treatment, we focused on the identification of an alternative E_2_ replacement regimen that could improve cognition and induce ischemic protection in females. In the current study we test the efficacy of a repetitive bolus of E_2_ (every 48 or 72 hours) on hippocampus-dependent learning, memory and ischemic neuronal damage in ovariectomized rats. The selection of timing for periodic E_2_ administration is based on our previous finding that an exogenous bolus of E_2_ 48/72 hours prior to ischemia reduced hippocampal damage in ovariectomized rats [6]. In this study we also demonstrate that E_2_ pretreatment-mediated ischemic protection occurs via activation of cyclic-AMP response element binding protein (CREB) [6].

Estradiol-17β-mediated CREB phosphorylation occurs via ligand-activated estrogen receptor subtypes alpha (ER-α) and beta (ER-β) in neurons [6]–[8]. Estrogen receptor(s) activation has been implicated to protect neurons from ischemic damage; however, there exists a discrepancy between which subtypes of estrogen receptor(s) are needed to confer beneficial effects of estrogen on brain [9]–[13]. Accumulating evidence also supports a key role for estrogen receptor(s) in hippocampus-dependent memory and cognition. Based on above the literature and our findings, we hypothesized that repetitive periodic E_2_ pretreatments provide neuroprotection against cerebral ischemia in ovriectomized rats, and that estrogen receptor(s) (ERs) are required to mediate the beneficial effects of periodic E_2_ on the hippocampus of ovariectomized rats. We also hypothesized that long-term periodic E_2_ or ER(s) agonist treatment improves learning and memory in ovariectomized rats. We tested this hypothesis using a rat model of global cerebral ischemia. The global cerebral ischemia is the consequence of cardiac arrest that causes delayed neuronal death in the hippocampal cornu ammon 1 (CA1) region [14]–[16].

## Materials and Methods

All animal procedures were carried out in accordance with the Guide for the Care and Use of Laboratory Animals published by the U.S. National Institutes of Health and were approved by the Animal Care and Use Committee of the University of Miami.

### *In vitro*

**Figure 1:**
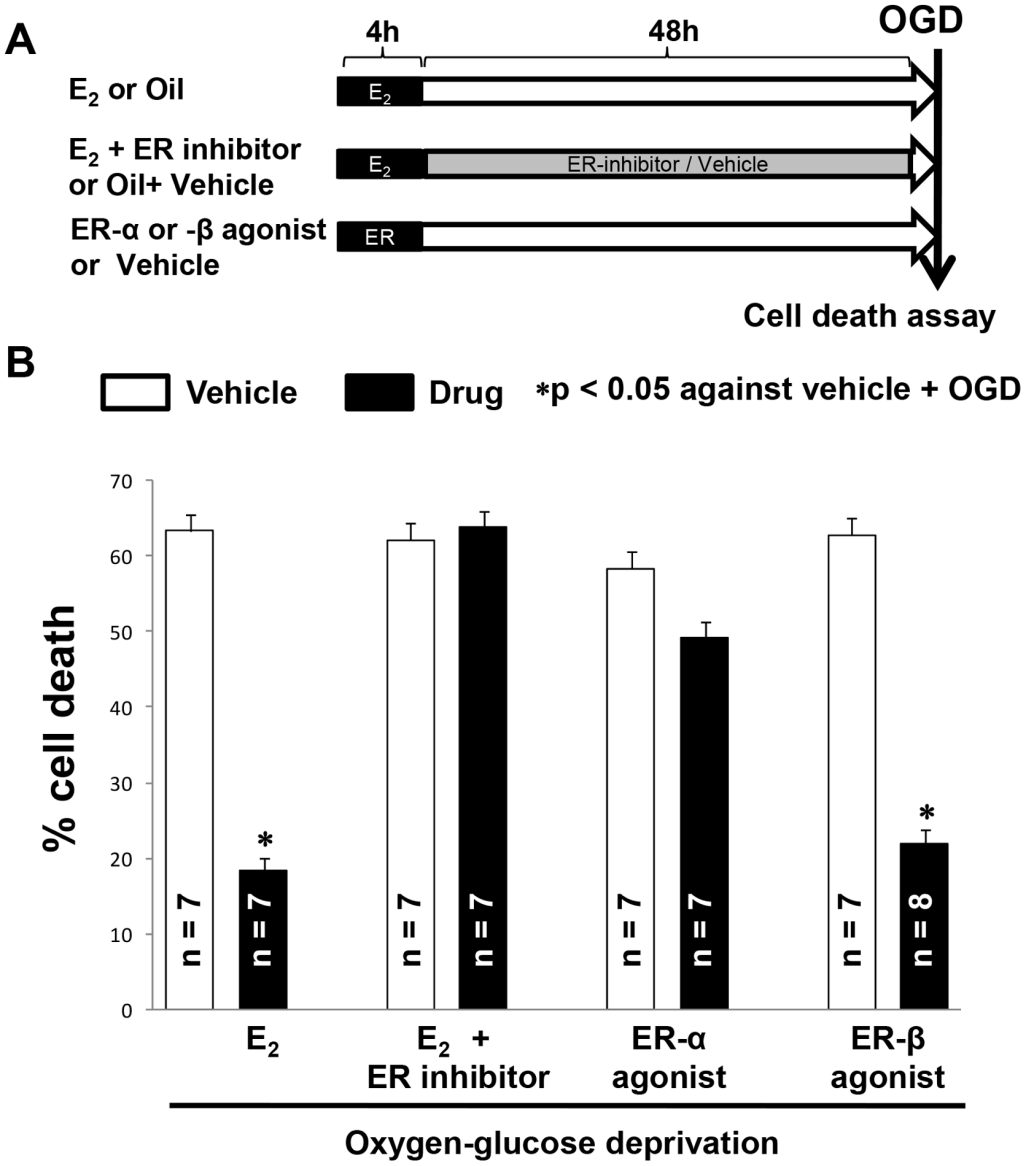
17β-estradiol-induced neuroprotection required estrogen receptor subtype beta. (**A**) Experimental design. (**B**) Histograms depict % of propidium iodide fluorescence values measured 24 hr following oxygen-glucose deprivation (OGD) in the experimental groups.

Organotypic hippocampal slice cultures were prepared as described previously [17]. Briefly, Sprague-Dawley female rats (9–11 days old) were anesthetized by intraperitoneal ketamine injection (1.0 mg). Animals were decapitated and the brains quickly removed. Transverse slices (400 µm) were dissected from the hippocampi and placed in Gey's Balanced Salt Solution (Sigma) supplemented with 6.5 mg/ml glucose at 4°C. After one hour, two slices were placed onto one 30 mm diameter membrane insert (Millicell-CM, Millipore) and inserts were transferred to six-well culture plates with 1 ml of culture medium per well [17]. The slice cultures were maintained (incubator equilibrated at 36°C, 95% O_2_, 5% CO_2_, and humidity 100%) for 14–15 days before use in one of the following experiments:

The role of ERs was determined by exposing slices to the medium containing E_2_ (1 nM; for 4 h) followed by slices being switched to medium containing ERs inhibitor (ICI 182780; 1 µM; DMSO) for 48 hrs (Figure 1A for experimental design). At the end of inhibitor treatment, slices were exposed to 40 min of oxygen-glucose deprivation (OGD) as described previously [17]. To further characterize the role of estrogen receptor subtypes, slices were either exposed to estrogen receptor subtype alpha (1,3,5-tris(4-hydroxyphenyl)-4-propyl-1H-pyrazole (PPT; 25 nM in DMSO) or beta 2,3-bis(4-hydroxyphenyl) propionitrile; DPN (40 nM in DMSO) selective agonist for 4 hr. Forty eight hours later, the slices were exposed to 40 min of oxygen-glucose deprivation. Assessment of neuronal cell death was conducted using the propidium iodide (PI) staining technique as described previously [17]. Propidium Iodide fluorescence images were obtained using a SPOT CCD camera and were digitized using SPOT advanced software. The percentage of relative optical intensity was used as an index of cell death.

### *In vivo:* Estrous Cycle Monitoring and Procedure of Ovariectomy (OvX)

Female Sprague-Dawley rats weighing 290±20 g were used for the study. Daily vaginal smears of female rats were collected (between 9∶00 and 10∶00 am) and identification of cell types was made microscopically [18]. Only rats showing at least three consecutive normal period (4 day) estrous cycles were used for experiments. Ovariectomy was performed on rats during the diestrus stage of estrous cycle as described previously [6].

### Knockdown of Hippocampal ER-β

**Figure 2:**
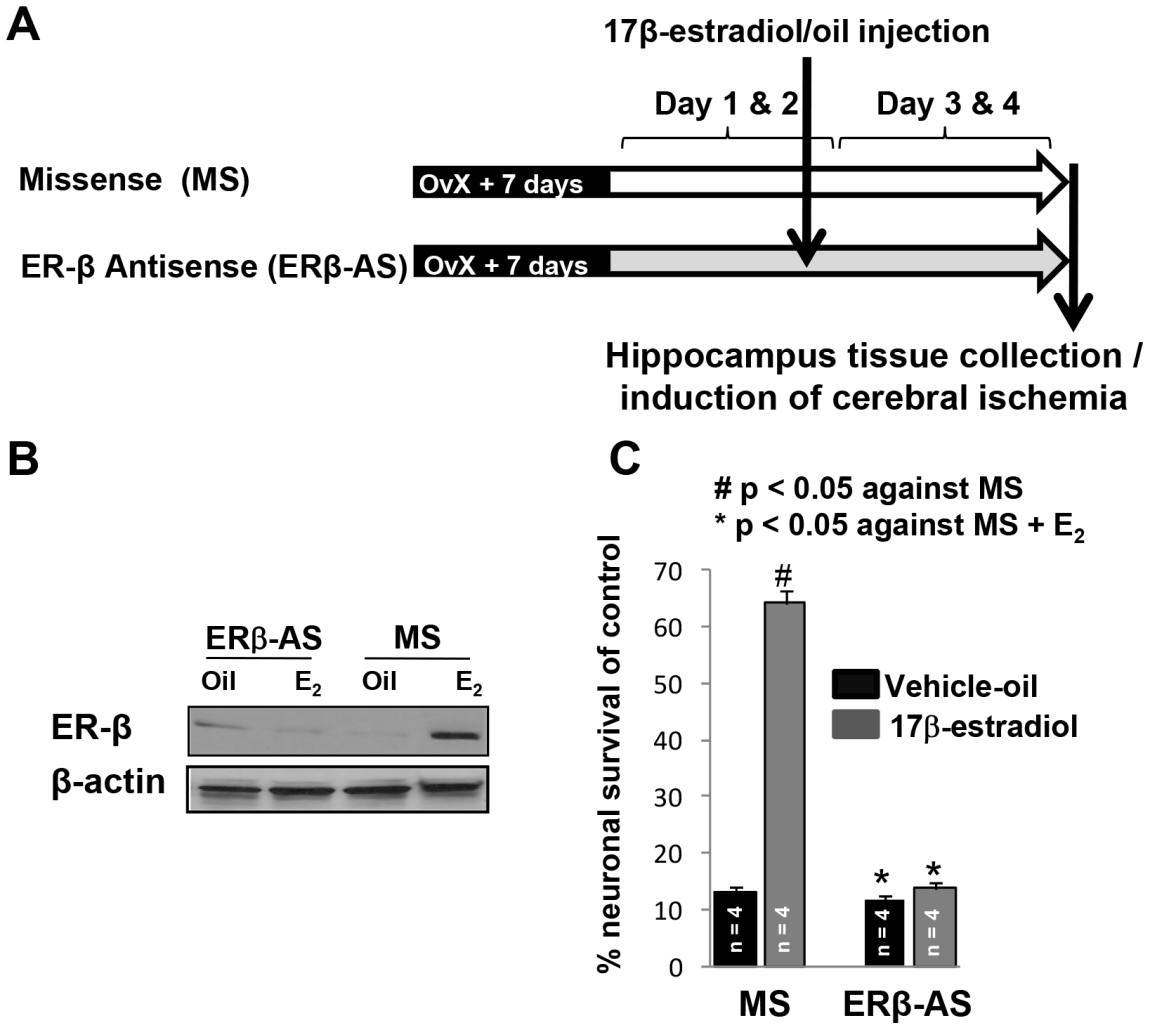
Silencing of ER-β inhibits 17β-estradiol-mediated hippocampal protection from cerebral ischemic damage. (**A**) Experimental design. (**B**) Representative immunoblots showing the protein levels of ER-β in the hippocampus of female rats. (**C**) Rats were treated with ER-β-antisense (AS) or missense (MS) by bilateral cerebroventricular infusion every 24 h for 4 days and E_2_/oil was administered on the second day of antisense treatment. Rats were exposed to cerebral ischemia 48 h after E_2_ treatment and 7 days later brains were examined for histopathology. The graph shows a percentage of live neurons in the hippocampal CA1 after cerebral ischemia.

We knocked down ER-β in the hippocampus using ER-β antisense oligodeoxynucleotide (AS) infusion (ER-β-AS), as described in our previous publication [19]. The antisense sequences used in this study were 5′-CATGGTCATGGTCAG-3′ for ER-α; 5′-GAATGTC ATAGCTGA-3′ for ER-β [20]. The same dose of a scrambled missense oligo (MS; 5′-ATCGTGGATCGTGAC-3′) was used as control. Integrated DNA Technologies prepared these sequences (Coralville, Iowa). The ER-β-AS or scrambled missense oligo (MS; 10 nmol of AS/MS) was administered by bilateral cerebroventricular infusion every 24 hrs for 4 days in the hippocampus of ovariectomized rats (Figure 2A). The delivery of antisense to hippocampal cells using this mode of delivery was confirmed in our previous study using immunofluorescence and immunoblotting approaches [19]. To stimulate estrogen signaling, rats were injected with a single bolus of E_2_ (5 µg) on the 2^nd^ day (48 hrs prior to sacrificing the rats) of AS treatment. We selected the 48 hour duration based on our previous study demonstrating that a single bolus of E_2_ phosphorylated CREB protein in the hippocampus of female rats after 48 hrs [6]. Following the last infusion of ER-β-AS or scrambled missense, rats were further divided in to two groups each containing ER-β-AS or scrambled missense treated rats. One group of rats was sacrificed and hippocampal tissue was collected to confirm inhibition of ER-β by Western blotting. While rats belongs to second group were exposed to cerebral ischemia and histopathological analysis was performed 7 days later.

### Periodic 17β-estradiol Treatment

**Figure 3:**
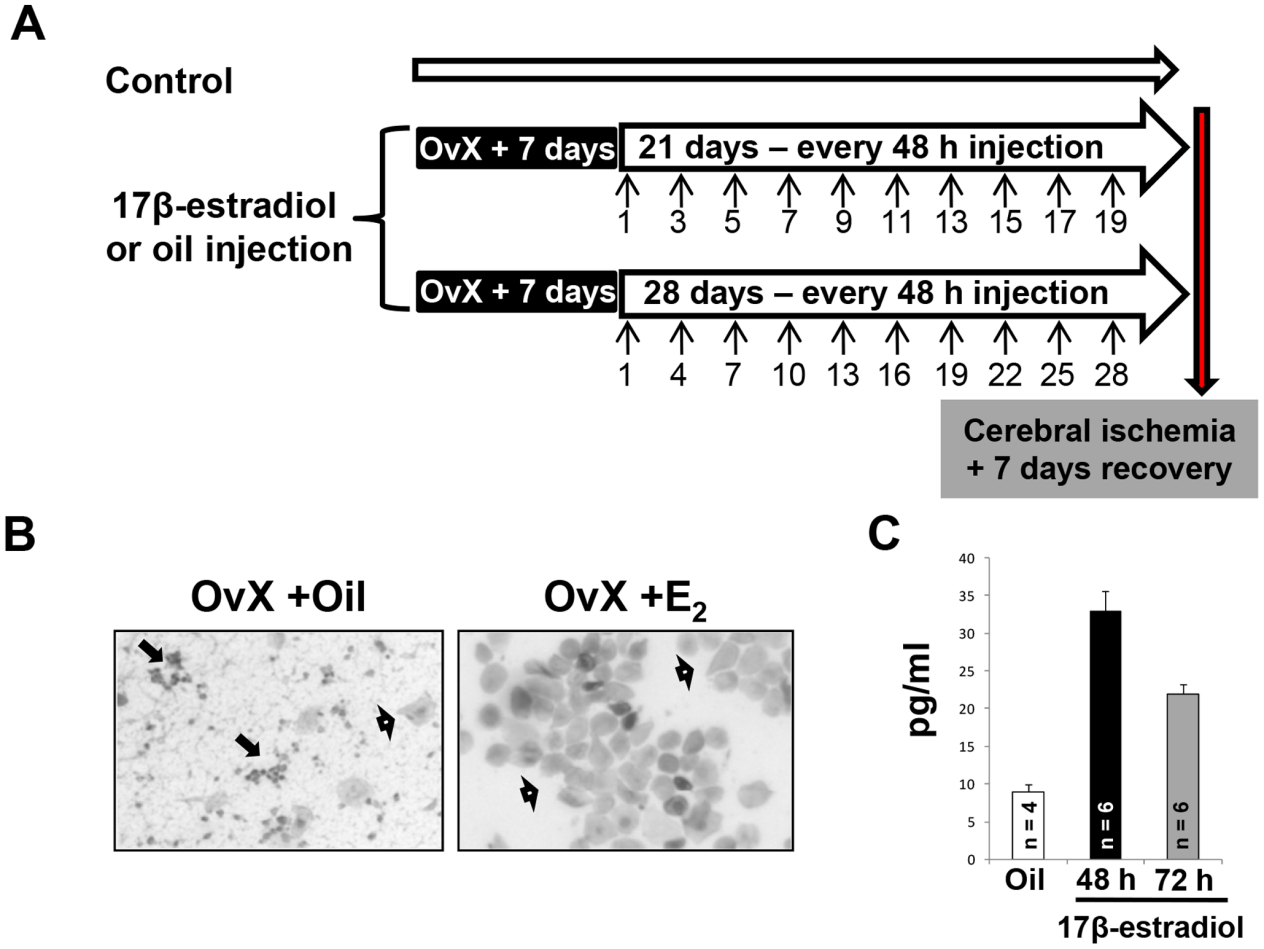
Diagram of experimental design and physiological parameters in periodic 17β-estradiol treated OvX female rats. (**A**) The OvX rats were given injections of 17β-estradiol or oil at 48 or 72 h intervals. Arrows indicate days that the rats received injections. Control rats were not OvX and did not receive any injections. (**B**) Pictures of vaginal histology in oil and E_2_ treated (48 hr) groups. Arrows show (<$>\raster(70%)="rg1"<$>) neurtrophils and (<$>\raster(75%)="rg2"<$>) superficial (acidophilic and basophilic). (**C**) The graph represents the plasma E_2_ levels 48/72 hrs after the last bolus of E_2_. (**D**) The graphs depict physiological data and arterial blood gases before and after cerebral ischemia.

Seven days after OvX, an injection schedule of 17β-estradiol (E_2,_ 5 µg/Kg, i.p.; dosage was based on our prior study [6]) or vehicle (oil) was started. Rats were injected ten times with E_2_/oil every 48 or 72 hrs (Figure 3A). Forty eight or 72 hrs following the last E_2_/oil treatment, rats were either exposed to cerebral ischemia or hippocampal tissue was collected for Western blotting. To confirm delivery and the effects of periodic E_2_ on OvX rats, vaginal lavage and blood were collected 48/72 hrs following the last injection. Vaginal smears were stained using Papanicolaou staining technique. The plasma concentration of estradiol was measured using a radioimmunoassay kit (Diagnostic Products Corporation, CA).

### Production of cerebral Ischemia

Cerebral ischemia was produced by 10 min of bilateral carotid occlusion and systemic hypotension (50 mm Hg). The details of this procedure remain same as described by us previously [6], [21]. Physiological variables (plasma glucose concentration, pH, PCO_2_, PO_2_ and mean arterial blood pressure (MABP)) were maintained normal before and after ischemia.

### Histopathology

After 7 days of reperfusion following ischemia, rats were anesthetized with isoflurane and perfused with FAM (a mixture of 40% formaldehyde, glacial acetic acid, and methanol, 1∶1:8 by volume). The head was removed and immersed in FAM at 4°C for 1 day. The coronal brain blocks were embedded in paraffin; coronal sections of 10 µm thickness were stained with hematoxylin and eosin. The stained sections were visualized at 40× magnification under a Nikon microscope equipped with a Sony CCD camera coupled to an MCID image analyzer (Imaging Research, St. Catherines, Ontario, Canada). For each animal, live neurons were counted in the CA1 region of each hippocampus by an investigator blinded to the experimental conditions. Coronal brain sections were made at the level of 3.8 mm from bregma. For each section, 18 fields were obtained, and three slides per rat were counted [6]. The data are presented as the mean count from these slides.

### Cell Fractionation and Western Blot Analysis

Hippocampi from rats exposed to the various treatment conditions described above were stored at −80°C. At the time of Western blot analysis, hippocampi were homogenized and cytosolic and nuclear fractions were prepared as described [6], [21]. The total, cytosolic or nuclear fractions were analyzed for protein content using the Bio-Rad protein assay kit. The proteins were separated by 12% SDS-PAGE. Equal amounts of protein from each group were run on the same gel and analyzed at the same time. Protein was transferred to Immobilon-P (Millipore) membrane and incubated with the primary antibody anti-β-actin (monoclonal; 1∶1000; Sigma), anti-lamin, anti-phospho-CREB (pSer133; rabbit polyclonal; 1∶1000; Cell Signaling Tech), and anti-ER-α/ER-β (rabbit polyclonal; 1∶500; Santa Cruz Biotech, CA); for the detection of β-actin, lamin, and pCREB and ER-β, respectively. β-actin and lamin were used as loading controls. Immunoreactivity was detected using enhanced chemiluminescence (Amersham-Pharmacia Biotech). Western images were digitized and subjected to densitometric analysis [6], [21]. An investigator blinded to the experimental conditions performed densitometric analysis.

### The Morris Water Maze

Ovariectomized rats exposed to periodic E_2_ (5 µg/Kg; vehicle oil) or ER-β agonist (DPN; 1 mg/kg; vehicle DMSO) treatment at 48 hrs for 21 days were used to monitor hippocampal-dependent learning and memory capabilities. The behavior testing paradigm lasted for seven days and rats were exposed to this paradigm starting day on 15 of the 21 day E_2_/ER-β agonist treatment. In a parallel experiment ER-β agonist treated OvX rats were exposed to cerebral ischemia and tested for neurobehavior seven days after induction of cerebral ischemia. An investigator blinded to the experimental conditions performed the behavior testing.

We performed a battery of behavioral tests, which was conducted over period of seven days. In this behavioral procedure, the rats were placed inside a small circular pool (122 cm diameter; 60 cm deep; filled with water at 21°C) with a platform located in the north-east corner. After a learning period of four days, the platform was removed. On day five the rats were allowed to swim freely and their tendency to return to the platform’s former location was recorded as an indicator of short-term memory. On days six and seven, their ability to find a new platform placed in a different location of the pool was assessed as an indicator of working memory. The working memory task was comprised of two sets of paired trials per day, for a total of 10 trials per day per rat, with the hidden platform and release location moved for every pair of trials. For each trial pair, an “exposure trial” was followed immediately by a “test” trial. As with the hidden platform task, animals were placed on the platform for 10 seconds if they failed to find the platform in 60 seconds. The animal’s ability to remember the platform location within each pair of trials was assessed, labeled as match in figures. The animal’s movement was videotaped with a CCD camera and analyzed with the Ethovision software program.

### Statistical Analysis

The data is presented as mean value ± SD. An analysis of variance (ANOVA) followed by a multiple comparison procedure (Bonferroni’s test) was used to analyze the statistical differences among groups. The data of the hidden platform and working memory was analysis by repeated measures ANOVA. In all cases, a *p* value less than 0.05 was considered statistically significant.

## Results

### Activation of ER-β Protects Hippocampal CA1 Neurons in an *in vitro* Model of Oxygen-Glucose Deprivation (OGD)

Our previous studies demonstrated that a single E_2_ pretreatment conferring post-ischemic neuroprotection required phosphorylation of CREB in both *in vivo* and *in vitro* models [6], [22]. Estrogen receptors mediate CREB phosphorylation; however, our studies did not identify which subtype(s) of estrogen receptor(s) is required for post-ischemic hippocampal protection after an E_2_ bolus. Therefore, we investigated the role of estrogen receptors by means of exposing E_2_ plus estrogen receptor(s) antagonist-treated slices to OGD. The PI fluorescence values of E_2_ and E_2_+ ERs inhibitor were 19±2 and 64±3 (p<0.05), respectively. The inhibition of ERs attenuated E_2_-mediated protection of CA1 neurons, suggesting involvement of ERs in E_2_-pretreatment-mediated neuronal protection from OGD injury. To further characterize the role of ERs, slices were treated with ER-α or ER-β agonist and exposed to OGD 48 hrs later. The PI fluorescence values of ER-α or ER-β agonist groups were 49±3 and 22±2, respectively. This data suggested that the activation of ER-α was not efficacious while ER-β agonist treatment significantly reduced post-OGD cell death (Figure 1).

### Silencing of ER-β Worsens Hippocampal Ischemic Damage in Ovariectomized Rats

The above results demonstrated that inhibition of estrogen receptors attenuated, and that ER-β activation protected neurons from OGD injury. Therefore, we tested the hypothesis that the ER-β silencing abolishes an E_2_ bolus-conferred neuronal protection in OvX rats. Using an antisense approach we knocked down ER-β in hippocampus of OvX rats. Forty-eight hrs prior to induction of cerebral ischemia, rats were treated with an E_2_/oil bolus. Forty-eight hrs after the bolus of E_2_/oil, a batch of rats was randomly selected to confirm knockdown of ER-β in hippocampus. First we confirmed by Western blot analysis the efficacy of the ER-β-AS in knocking down β in the hippocampus (Figure 2B). Rats exposed to an ischemic episode were used for histopathological analysis of the hippocampus. The number of live neurons per slice in the CA1 hippocampal region in control rats was 1100±68 (100%; n = 3) and ischemic insult to MS+oil-treated OvX females decreased the live neuronal count to 13% (142±14, p<0.001; Figure 2C). An estradiol bolus to MS exposed OvX rats increased the number of normal neurons to 64% (705±28). However, an E_2_ bolus to ER-β-AS-exposed OvX rats resulted in only 13% (150±9) of live neurons. These results suggested that the ER-β-AS attenuated E_2_-mediated neuroprotection against cerebral ischemia, underscoring a role for ER-β.

### Periodic 17β-estradiol Replacement Protects CA1 Neurons from Ischemia

In our previous study we observed that an exogenous bolus of E_2_ 48 or 72 hrs prior to cerebral ischemia protected hippocampus in OvX rats [6]. As a continuation, in the present study we tested the hypothesis that repetitive periodic E_2_ exposure prior to an ischemic episode reduces post-ischemic CA1 hippocampal neuronal loss in OvX rats. We tested this hypothesis by treating OvX rats with ten boluses of E_2_ at 48/72 hrs (Figure 3A describes an experimental design). To confirm the bio-availability of E_2_, we examined the vaginal histology and plasma E_2_ levels 48/72 hrs after the last bolus of E_2_. The presence of superficial (acidophilic and basophilic) cells in the vaginal smears of periodic E_2_-treated OvX rats confirmed the effect of E_2_ (Figure 3B)_._ Furthermore, plasma E_2_ levels in the 48 and 72 hrs periodic E_2_-treated groups were 33±3 and 22±2, which were higher than OvX (9±1; p<0.005) rats (Figure 3C).

**Figure 4:**
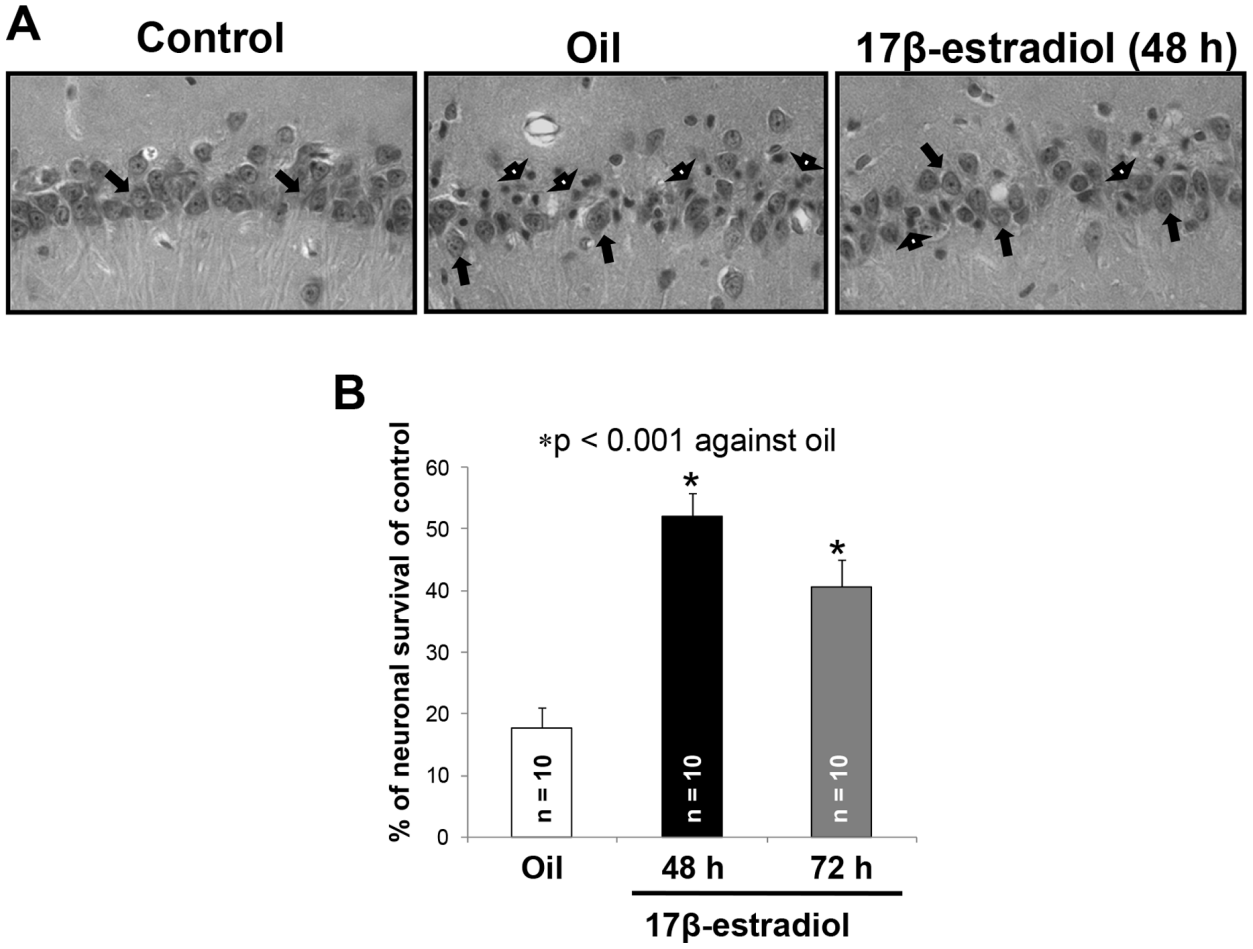
Periodic 17β-estradiol (E_2_) treatment every 48 hrs protects hippocampal neurons from cerebral ischemic damage. (**A**) Representative histological images in the hippocampal CA1 region (40×). Arrows show (<$>\raster(70%)="rg1"<$>) live and (<$>\raster(75%)="rg2"<$>) dead neurons. (**B**) Percent neuron survival in the CA1 region of the rat hippocampus.

Forty-eight or 72 hours after the last E_2_ treatment, rats were exposed to an episode of cerebral ischemia. All the physiological variables (pH, PCO_2_, PO_2_ and mean arterial blood pressure (MABP)) remained unchanged throughout the surgical procedure for cerebral ischemia induction in all the experimental groups. Additionally, these parameters were not different between groups. Histopathology was carried out 7 days following ischemia. The number of live neurons per slice in the CA1 hippocampal region in control rats (sham ischemia) was 1100±45. Ischemia in oil-treated OvX rats decreased the number of surviving neurons by 82% (192±11). Periodic E_2_ treatment increased the number of live neurons by 52% (568±13, every 48 h) and 41% (446±15, every 72 h) compared to the OvX group (p<0.05). Since the previous results did not show a significant difference in percent of live neurons between the 48 and 72 hrs-treated groups, we selected periodic E_2_ treatment at 48 h for subsequent experiments (Figure 4).

In an additional control experiment, female rats we exposed to the sham-OvX procedure and exposed to an ischemic episode seven days later. The number of live neurons in CA1 region of sham-OvX rats was higher compared to oil-treated OvX rats, confirming that the role of endogenous circulating E_2_ is similar to that presented in our previous study, thus the data is not presented here [6].

### Periodic 17β-estradiol or ER-β Agonist Treatment Increased CREB Phosphorylation in the Hippocampus of OvX rats

**Figure 5:**
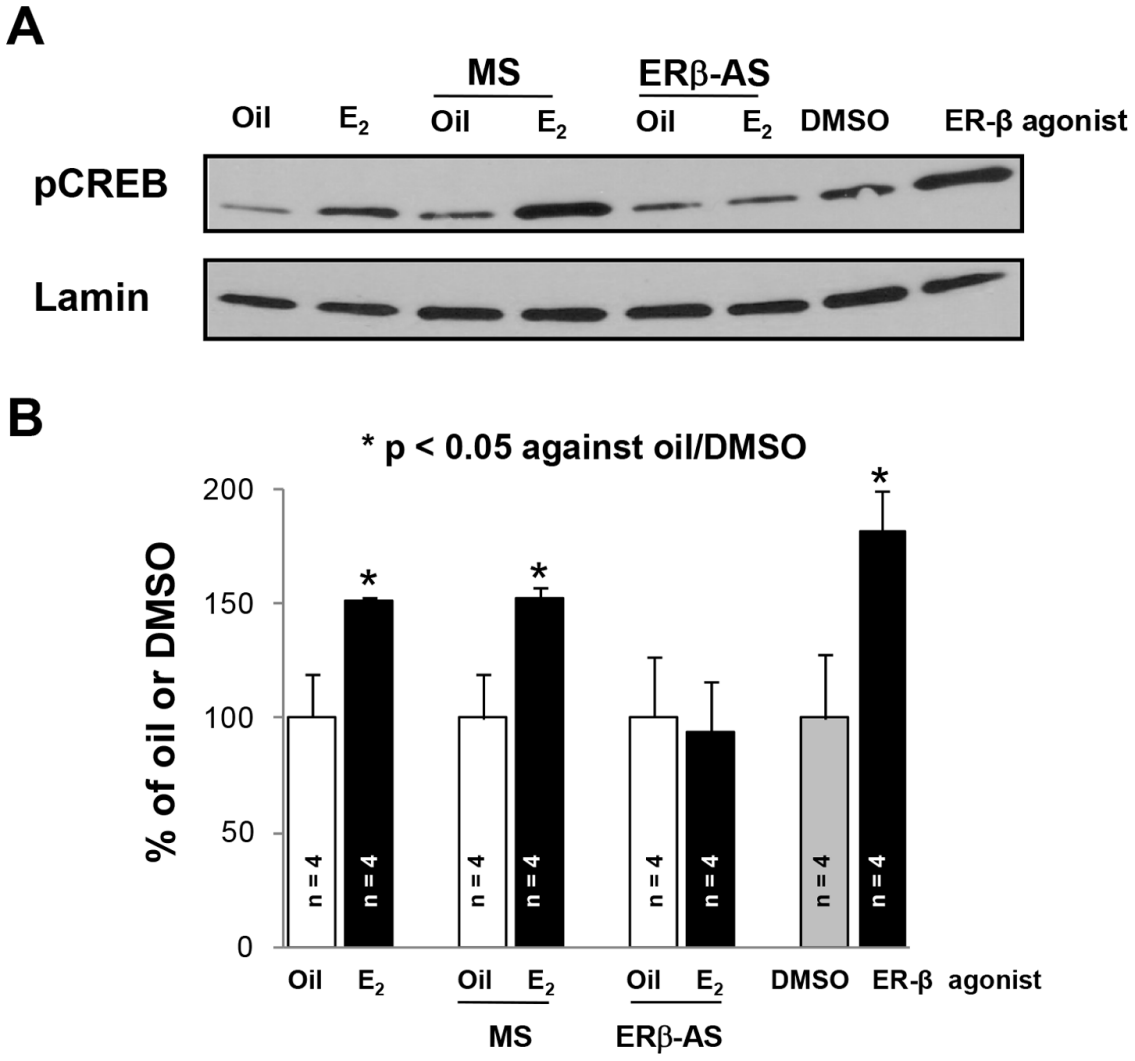
Estrogen mediates phosphorylation of CREB via activation of ER-β. (**A**) Representative immunoblots showing the protein levels of pCREB in the hippocampus for different experimental conditions. (**B**) The graph depicts densitometric analysis of scanned Western blots.

Activation of ER-β phosphorylates CREB and an E_2_ bolus conferred post-ischemic hippocampal protection requires pCREB [6], [22], [23]. Therefore, we tested the hypothesis that the periodic E_2_ treatments mediate CREB phosphorylation in hippocampus of OvX rats. Western blotting demonstrated that the periodic E_2_ treatment increased hippocampal pCREB protein level by 53% as compared to the oil treated group. Silencing of hippocampal ER-β reduced E_2_-mediated pCREB protein levels by 53% as compared to the MS+E_2_-treated group (p<0.05; Figure 5).

Because we observed that the periodic E_2_ treatment increased pCREB protein levels, we next tested the effects of periodic ER-β activation on pCREB protein level. Periodic activation of ER-β increased hippocampal pCREB protein level by 81% as compared to the DMSO-treated group. These results suggested that periodic E_2_-induced phosphorylation of CREB is mediated via ER-β and periodic ER-β activation simulates the effect of periodic E_2_ administration on CREB phosphorylation in hippocampus of OvX rats (Figure 5).

### Periodic 17β-estradiol or ER-β Agonist Replacement Improved Learning and Memory

**Figure 6:**
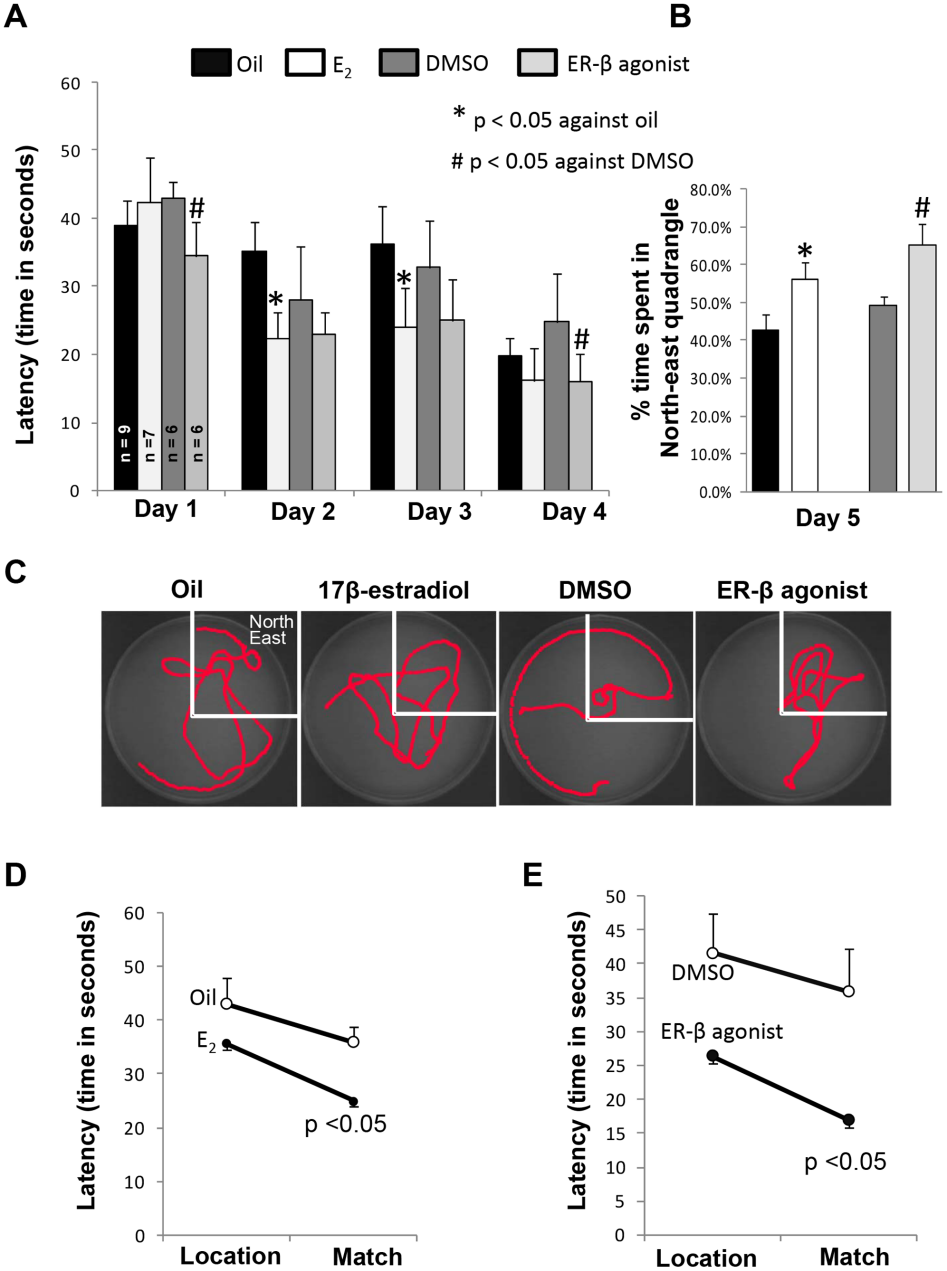
Periodic 17β-estradiol or ER-β agonist pretreatment improves hippocampus-dependent spatial learning and memory in OvX rats. (**A**) **Acquisition/training/learning:** Spatial learning acquisition water maze performance in different experimental groups. Latency to platform was measured for four days and each day four trials were performed. Latency to platform is plotted for the average of each day of testing. (**B & C**) **Probe Trail: B:** Bar graph shows percent of time spent in the north-east quadrant for each experimental group. **C:** Representative traces indicating the sample paths of the rats from probe trials. (**D**) **Working Memory Task:** Location-match pair of trials was carried out with the hidden platform in a novel location within the water tank. Note the significant difference in the latency to match the location in E_2_ and ER-β agonist treatment compared with their respective vehicle - treated groups.

Next, we tested the hypothesis that periodic E_2_ or ER-β agonist treatment improves learning and memory in OvX female rats. Ovariectomized rats were exposed to periodic E_2_/ER-β agonist treatment at 48 hr intervals for 21 days. Behavioral assessments started on day 15 of the 21 day treatment. The periodic E_2_/ER-β-treated rats showed significantly lower latencies in finding the submerged platform in comparison to the vehicle (oil or DMSO) treated OvX rats (Figure 6A). The probe trial conducted on the day fifth of behavioral tests demonstrated that the periodic ER-β agonist-treated OvX rats spent significantly greater amounts of time in the quadrant where the submerged platform was located as compared to the vehicle group (Figure 6B). The last part of the behavior assessment tested working memory. We observed a significant difference in the latency required to match the location by the recipients of the E_2_ and ER-β agonist treatment in comparison to the vehicle- treated groups (p<0.05; Figure 6C).

### Periodic ER-β Agonist Replacement Improved Post-ischemic Learning and Memory

**Figure 7:**
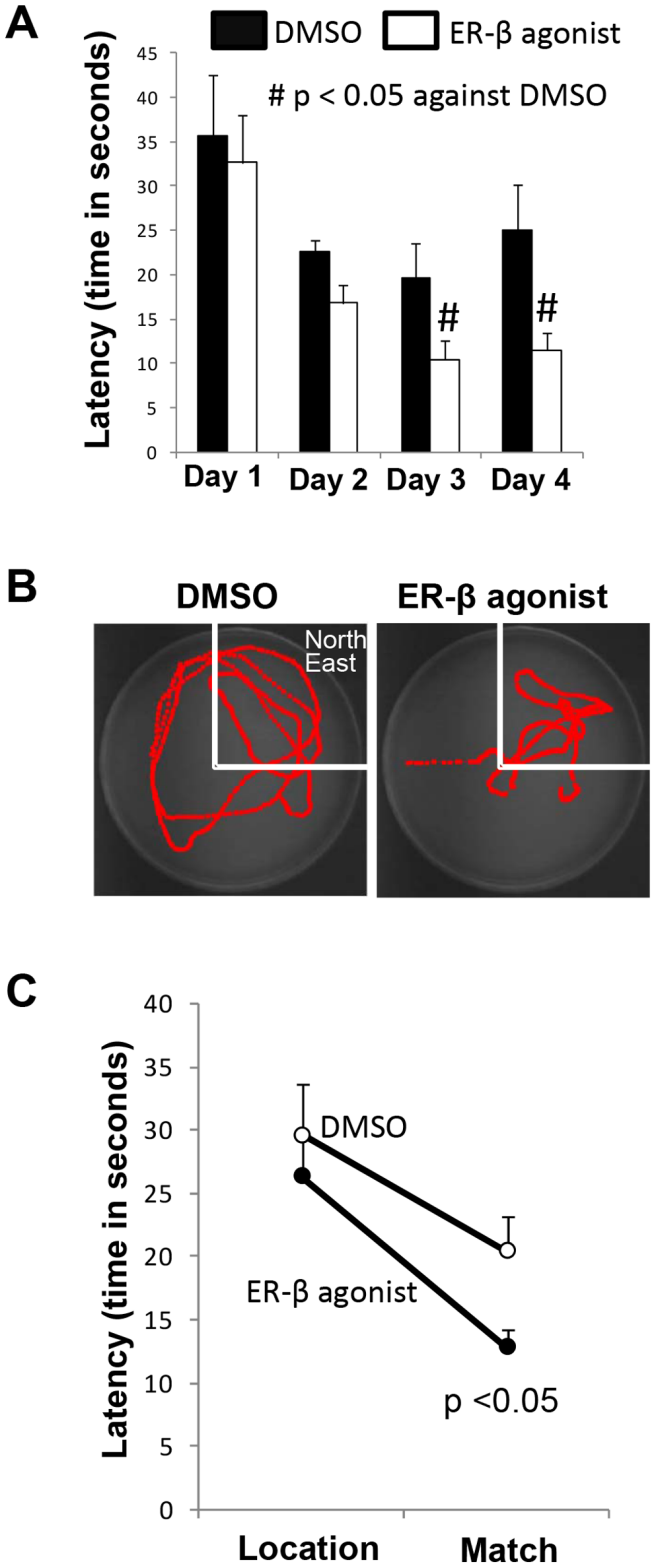
Periodic ER-β agonist pretreatment improves post-ischemic hippocampus-dependent spatial learning and memory in OvX rats. (**A**) Spatial learning acquisition water maze performance seven days after cerebral ischemia in ER-β agonist/vehicle treated rats. (**B**) Representative traces indicating the sample paths of the rats from probe trials. (**C**) Location-match pair of trials was carried out with the hidden platform in a novel location within the water tank. Note the significant reduction in the latency to match the location in ER-β agonist treated rats which underwent ischemia.

Finally, we tested hypothesis that periodic activation of ER-β improves post-ischemic hippocampus-dependent learning and memory. Periodic ER-β agonist/vehicle treated OvX rats were exposed to cerebral ischemia and behavioral assessment was conducted seven days later. We observed significantly lower latencies in finding the submerged platform in periodic ER-β-treated group in comparison to the vehicle (DMSO) treated group after cerebral ischemia (Figure 7A). The probe trial conducted on the day fifth of behavioral tests demonstrated that the OvX rats exposed to ischemia after periodic ER-β agonist-treatment spent significantly greater amounts of time in the quadrant where the submerged platform was located as compared to the vehicle group (Figure 7B). In addition to monitoring time spent in the quadrant, the swim speed was analyzed and no difference was noted between two groups. We also observed similar improvement in the last part of the behavior assessment which tested working memory. The latency required to match the location was significantly less in the ER-β agonist treated group than in the vehicle- treated groups after ischemia (Figure 7C).

## Discussion

This study demonstrated that long-term periodic E_2_ replacement significantly reduced post-ischemic hippocampal injury in OvX rats. Silencing of ER-β in the hippocampus of OvX rats using an antisense approach attenuated E_2_-mediated ischemic protection, which suggests that ER-β plays a key role in mediating the beneficial effects of periodic E_2_ treatments. Periodic activation of ER-β significantly improved hippocampal-dependent learning and memory. Overall, our results suggest that long-term periodic E_2_ treatment improves post-ischemic outcome and cognition via ER-β intracellular signaling in the hippocampus of OvX female rats.

Consistent with our findings as detailed above, a previous study showed that chronic continuous E_2_ treatment prior to cardiac arrest was neuroprotective and was mediated through ER-β [9]. Continuous pretreatment with the ER-β agonist, by contrast, could not reduce infarct size or sensorimotor function in rats exposed to middle-cerebral artery occlusion, and hence suggested no role for ER-β [10]. Studies from other groups demonstrated that E_2_-induced ischemic protection required ER-α activation [11]–[13]. This contradiction suggests that both estrogen receptors are crucial for neuronal survival, although the mechanisms of neuroprotection governed by these receptors might be different and dependent on dosage as well as the paradigms of E_2_ treatment. A recent review also suggested that estrogen receptor-dependent mechanisms of neuroprotection could vary depending on the model used, the level of estrogen administered and the mode of delivery of the steroid (see review [24]). Based on the aforementioned idea and current findings, we propose that the periodic E_2_ bolus mediated the beneficial effects on the hippocampus via ER-β. However, a caveat of this study is that it does not test the possibility that continuous versus periodic E_2_ administration could initiate potentially different mechanisms. The exact cascade of ER-β-mediated intracellular signaling responsible for post-ischemic neuroprotection also remains to be elucidated. In the following paragraphs we discuss possible mechanisms by which ER-β activation prevents ischemic neuronal loss after periodic E_2_ treatment.

Mitochondrial dysfunction remains the main cause of neuronal death after ischemia, and one of estrogen’s putative effects involves protection of mitochondrial function [25]–[27]. The mechanism by which E_2_ preserves mitochondrial function is not well understood. However, studies demonstrating the presence of estrogen receptors on mitochondria suggest their role in the regulation of mitochondrial function [28]–[34]. Briefly, the mitochondrial respiratory chain consists of four multi-subunit complexes (I–IV) that, through electron transport reactions, generate a proton gradient for the F_1_F_o_-ATPase (complex V) to synthesize ATP [35]–[37]. Cytochrome c oxidase (complex IV) catalyzes the final step of the electron transfer chain, in which electrons are transferred from reduced cytochrome *c* to molecular oxygen [35]. In a recent study, we demonstrated that ER-β agonist treatment in isolated mitochondria significantly increased complex IV enzyme activity while ER-α agonist treatment failed to do so. This study confirmed the role of mitochondrial ER-β in regulation of complex IV function. Additionally, silencing of hippocampal ER-β lowered protein levels of mitochondria-encoded complex IV subunits 1, 2 and 3 and supported a role for ER-β in protein expression of the mitochondrial oxidative phosphorylation system [19]. Complex IV is a homodimer made up of thirteen subunits, three of which form the catalytic core as encoded by the mtDNA [35]. It is known that pCREB regulates gene expression of OXPHOS subunits by binding directly to the control region of mtDNA. [38]–[40]. Silencing of hippocampal ER-β reduced mitochondrial pCREB following E_2_ treatment, suggesting that the ER-β is essential for CREB phosphorylation [19]. Additionally, pCREB is also known to regulate expression of the pro-survival protein B-cell lymphoma-2 (Bcl-2). Bcl-2 stabilizes the mitochondrial membrane potential and prevents the release of mitochondrial cytochrome c, thus blocking activation of the caspase cascade and the onset of cell death [41], [42]. Estrogen treatment attenuates ischemia-induced caspase-3 activation as well as cytochrome *c* release from mitochondria in the hippocampus, thus protecting neurons from ischemic injury [2], [43]. Therefore, improved mitochondrial function, biogenesis and anti-apoptotic signaling offer a possible explanation for the observed significant improvement in post-ischemic neuronal survival in periodic E_2_-exposed OvX rats; however, this aspect remains to be investigated.

Estrogen receptor-beta (ER-β) is predominantly expressed in the hippocampus and synaptic ER-β is suggested to be a more responsive target to E_2._ Loss of synaptic function leads to neuronal cell death in the hippocampus after cerebral ischemia [44]. In hippocampus, E_2_-signaling regulates synaptic plasticity by pCREB [45]. The selective activation of ER-β increased protein levels of pCREB; thus, periodic E_2_ treatment might be responsible for preserving synaptic functions that could be a potential mediator of improved neuronal survival after an ischemic episode in OvX rats.

Estrogens have long been implicated in influencing cognitive processes, yet the roles of the estrogen receptor subtypes remained unclear until recently. It has been demonstrated that ER-β knockout mice treated with E_2_ show impairments in acquisition of a spatial reference memory, implying that ER-β plays a role in hippocampus-dependent cognition [46], a phenomenon now supported by accumulating evidence. Continuous activation of ER-β was found to improve hippocampus-dependent cognition in female mice [47]. In this context, the therapeutic potential of the ER-β-selective modulators is suggested for the prevention of climacteric symptoms and decline in brain responses induced by ovarian hormone loss in menopause [48]. The current study also demonstrates that periodic ER-β agonist treatment improves post-ischemic spatial learning and memory in young adult OvX female rats. Our use of periodic regimen of the ER-β agonist pretreatment could avoid serious side effects, such as breast and uterine cancers, since ER-β-selective agonist does not stimulate the proliferation of breast or endometrial tissues. [49]–[51]. By contrast, activation of ER-α with the ER-α-selective agonist (propyl pyrazole triol) is associated with proliferative responses in breast and uterine tissues [49]–[51]. Future studies using periodic ER-β-selective modulators could be safe and beneficial in prevention of age-related decline in cognition. Studies also demonstrate that the expression of estrogen receptors changes with aging. Therefore, the effects of periodic E_2_ or ER-β modulators on middle-aged or reproductively senescent females need further investigation and remain limitation of current study [52]. Another limitation of our study is that we could not monitor circulation plasma E_2_ levels continuously following periodic E_2_/ER-β agonist treatment. To develop periodic E_2_/ER-β agonist treatment regime that could mimic endogenous plasma E_2_ fluctuations we need to monitor plasma E_2_ levels continuously after bolus of E_2_/ER-β agonist.

Finally, our results demonstrated that the novel regimen of periodic 17β-estradiol treatment significantly improved ischemic outcome and cognition in OvX rats. The observed beneficial effects of periodic E_2_ were governed via its receptor subtype beta-mediated CREB pathway. ER-β has been implicated to regulate mitochondrial functions that might be potential mechanisms of post-ischemic cell survival, and warrant further investigation. These results also emphasize the need to investigate the periodic ER-β agonist treatment regimen to reduce cognitive decline and cerebral ischemia incidents/impact in post-menopausal women.
